# Cytokine-Induced Modulation of Colorectal Cancer

**DOI:** 10.3389/fonc.2016.00096

**Published:** 2016-04-19

**Authors:** Lukas F. Mager, Marie-Hélène Wasmer, Tilman T. Rau, Philippe Krebs

**Affiliations:** ^1^Institute of Pathology, University of Bern, Bern, Switzerland; ^2^Graduate School for Cellular and Biomedical Sciences, University of Bern, Bern, Switzerland

**Keywords:** colorectal cancer, inflammation, cytokine, tumor microenvironment, biomarker

## Abstract

The emergence of novel immunomodulatory cancer therapies over the last decade, above all immune checkpoint blockade, has significantly advanced tumor treatment. For colorectal cancer (CRC), a novel scoring system based on the immune cell infiltration in tumors has greatly improved disease prognostic evaluation and guidance to more specific therapy. These findings underline the relevance of tumor immunology in the future handling and therapeutic approach of malignant disease. Inflammation can either promote or suppress CRC pathogenesis and inflammatory mediators, mainly cytokines, critically determine the pro- or anti-tumorigenic signals within the tumor environment. Here, we review the current knowledge on the cytokines known to be critically involved in CRC development and illustrate their mechanisms of action. We also highlight similarities and differences between CRC patients and murine models of CRC and point out cytokines with an ambivalent role for intestinal cancer. We also identify some of the future challenges in the field that should be addressed for the development of more effective immunomodulatory therapies.

## Introduction

Colorectal cancer is the second and third most common malignant disease in women and men, respectively (1). Estimations predict that there will be over 750,000 new cases and more than 350,000 deaths from colorectal cancer (CRC) in developed countries in 2015 alone [reviewed in Ref. (2)]. A large number of genetic aberrations have been identified that underlie CRC (3, 4). Somatically altered genes or polymorphisms that are repeatedly found in CRC often affect the KRAS-, MYC-, Wnt-, mitogen-activated protein kinase (MAPK)-, or TGF-β/bone morphogenetic protein (BMP)-signaling pathways, lamina structural proteins or components of the DNA repair machinery [reviewed in Ref. (2, 5)]. Recently, several factors have received increased attention that are distinct from tumor cells and that substantially contribute to cancer progression. These include non-cancerous cells in the vicinity of the tumor, which are commonly referred to as tumor microenvironment or stroma, and the microbiota. Non-malignant cells in the tumor mass comprise (myo)fibroblasts, endothelial cells, and immune cells (6). Although fibroblasts and endothelial cells can promote CRC (7–9), the immune infiltrates in the tumor microenvironment appear to differently modulate CRC development, depending on their nature. Initial studies reporting a correlation between the inflammatory cell pattern in CRC tumors and the prognosis (10, 11) have been further elaborated by Galon and colleagues into the “Immunoscore,” a classification that has improved the prognostication of CRC development (12–14). In essence, this work established that analysis of the composition of the immune infiltrates enhances the accuracy of prognostic information and predictability of response to therapy. Therefore, it provides complementary information to the traditional American Joint Committee on Cancer (AJCC)/Union for International Cancer Control (UICC)-TNM classification applied for more than 80 years. The Immunoscore analyzes the regional density of all (CD3^+^) T cells, including CD8^+^ cytotoxic T cells, in human CRC. This allows in a next step to associate T cell differentiation with CRC progression; e.g., a T helper 1 (T_h_1) signature correlates with better disease-free survival, whereas a T_h_17 signature is predictive of the opposite (15).

A central feature of activated immune cells is the production and release of growth factors and cytokines that modulate the inflammatory milieu in tumor tissues. Systemic and local changes in the cytokine profile have been shown in CRC (16–18). Recent work indicates that multiple pro-tumorigenic and also anti-tumorigenic cytokines are differently expressed in distinct CRC tissues (19). Therefore, it is critical to study the specific contribution of individual cytokines to CRC development, progression, and patient survival.

Here, we review the current knowledge on cytokines known to modulate intestinal tumor development. Each listed cytokine is systemically presented according to the following scheme: (1) role as a biomarker in samples of CRC patients, (2) phenotype of knockout or transgenic mice in experimental models of CRC or intestinal inflammation, (3) tissue of origin or type of the secreting cells, (4) nature of the receptor-expressing cells in the tumor stroma, (5) biological effect on human/murine primary cells or cell lines, and (6) molecular mechanisms and pathways activated upon receptor engagement.

**Figure 1:**
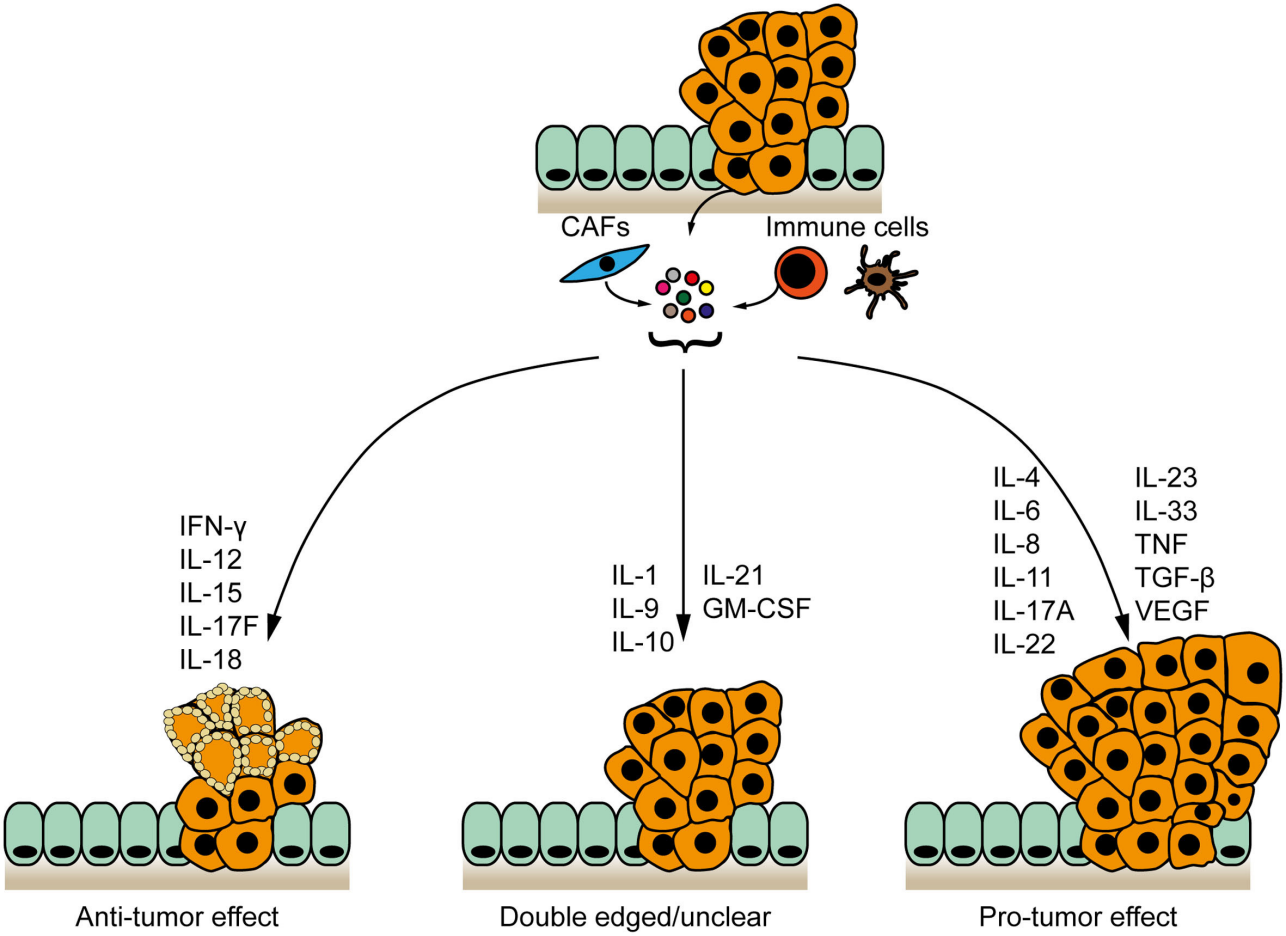
**Cytokine networks in the pathogenesis of colorectal cancer**. Cytokines expressed by tumor and/or stromal cells cluster to form networks with anti-tumor, pro-tumor, or bivalent properties. IFN-γ, interleukin-12 (IL-12), IL-15, IL-17F, and IL-18 inhibit CRC development. IL-4, IL-6, IL-8, IL-11, IL-17A, IL-22, IL-23, IL-33, TNF, TGF-β, and VEGF are pro-tumorigenic. The contribution of IL-1, IL-9 IL-10, IL21 and GM-CSF to intestinal cancer remains unclear.

As a synthesis, we highlight two major, functionally different inflammatory networks: a network of inflammatory mediators driving (antigen)-specific anti-tumor immunity to inhibit tumor development (19, 20) and a nexus of cytokines supporting chronic, unspecific, pro-tumorigenic inflammation in the CRC microenvironment (Figure 1), which is associated with a disruption of the intestinal barrier and invasion by microbial products (21). It is likely that the balance between these two opposite inflammatory networks within the tumor stroma determines the course of CRC development. We also propose that these different groups of cytokines may be used as biomarkers to strengthen the diagnostic based on the Immunoscore, and that they represent targets for the design of therapeutic approaches.

## Interleukin-1

The role of IL-1 in CRC is controversial. *IL1B* transcripts are increased in tumor biopsies of patients with metastatic CRC (22) and polymorphisms in IL-1 receptor antagonist (IL-1RA) (encoded by *IL1RN*) may be associated with CRC (23). IL-1β is activated by caspase-mediated cleavage subsequent to the activation of the leucine-rich repeat (NLR) inflammasome as a response to cell stress or infection. The NLR inflammasome plays an important role in the development of colitis-associated cancer (CAC). *In vivo* blockade of IL-1β using recombinant IL-1RA significantly decreased tumor development in the AOM/DSS mouse model of CAC (24), indicating a pro-tumorigenic role of IL-1β in this setting. IL-1β is expressed by tumor-associated macrophages (TAMs) (25) and neutrophils (24). IL-1β can act on intestinal epithelial cells (IECs) (24) and directly on tumor cells (25) to induce their proliferation. IL-1β also promotes the recruitment of myeloid-derived suppressor cells (MDSCs) to tumors, which supports cancer progression (26–28). Furthermore, colon cancer cell-derived IL-1α may upregulate angiogenesis by modulating stromal cells within the tumor microenvironment (29).

Interleukin-1 binds to either IL-1Rα (encoded by *IL1R1*) that has a long cytoplasmic domain to relay signaling or to IL-1Rβ (encoded by *IL1R2*) that may act as a decoy receptor. Upon IL-1 binding, IL-1Rα forms a complex with IL-1 receptor accessory protein (encoded by *IL1RAP*). This induces the recruitment and activation of the IRAK and TRAF6 adapter molecules and the activation of nuclear factor κB (NF-κB), JNK, AP-1, and p38 MAPK pathways [reviewed in Ref. (30)]. Furthermore, IL-1β induces the activation of the Wnt signaling pathway by phosphorylation of GSK3β (25). Importantly, all these signaling pathways are key for intestinal tumorigenesis (31–33), thereby further supporting the central role of IL-1 for CRC pathogenesis.

## Interleukin-4

Interleukin-4 is overexpressed in early events of CRC development, including hyperplastic polyp, adenoma, and serrated adenomas, whereas in adenocarcinomas, IL-4 levels are not elevated compared with normal mucosa (34). In addition, higher serum levels of IL-4 were found in CRC patients with distant metastases (M1) compared with patients without metastases (M0) (16). However, presence of a T_h_2 gene signature – comprising IL4, IL5, and IL13 – in human CRC does not appear to have a prognostic value (15).

In experimental animal models of CRC, *Il4*-deficient mice treated with AOM developed fewer tumors compared with wild-type (WT) mice (35). In the AOM/DSS model of tumorigenesis, signaling through IL-4 receptor α (IL-4Rα) promoted intestinal tumor growth (36). However, both IL-4 and IL-13 engage IL-4Rα, which prevents a clear estimation of IL-4 function in CRC in this study. To bypass this issue, Ingram et al. evaluated intestinal tumor formation in AOM-treated *Il13*^−/−^ mice, thus only allowing signaling of IL-4 trough IL-4Rα. They found that intestinal tumor development was markedly increased in *Il13*^−/−^ compared with WT mice (37), which further indicates a pro-tumorigenic effect of IL-4 in CRC.

T_h_2 and double-positive CD4^+^ CD8αβ^+^ αβ T cells as well as cancer-initiating cells are important sources of secreted IL-4 in CRC (38, 39). IL-4 signaling occurs through either the type I or type II IL-4 receptor. The type I IL-4 receptor complex is composed of IL-4Rα and the common gamma chain, whereas the type II IL-4 receptor consists of the IL-4Rα and IL-13Rα1 subunits (40). Type I IL-4 receptor is predominately expressed on hematopoietic cells, whereas type II IL-4 receptor expression is high on transformed IECs (36). This expression pattern suggests that IL-4 may have a direct and an indirect effect on CRC development. Indeed, the proliferation of several CRC cell lines was increased after IL-4 stimulation (36). Furthermore, *in vitro* coculture of IL-4-secreting CRC-derived tumor-initiating cells with peripheral blood mononuclear cells (PBMCs) was found to inhibit the proliferation of these PBMCs, which could be restored upon addition of IL-4 blocking antibodies. This may serve as a mechanism for tumor-initiating cells to escape immune surveillance and in turn promote CRC progression (39).

Mechanistically, IL-4/IL-4 receptor engagement leads to signal transducer and activator of transcription (STAT)-6 phosphorylation in hematopoietic (41) and epithelial cells (42). Increased STAT6 phosphorylation in CRC tumors negatively correlates with survival in patients (43). Of note, IL-4 has also been shown to inhibit tumor growth and progression in other tissues, such as renal cancer (44) and glioblastoma (45). This was dependent on tumor-specific CD8^+^ T cells or associated with a marked eosinophil infiltrate, respectively. In addition, T_h_2 immune responses have been shown to induce IL-4- and eosinophil-dependent anti-tumor activity (46). Thus, IL-4 may have distinct functions, depending on the tumor environment. However, in CRC IL-4 rather appears to drive tumor development.

## Interleukin-6

Interleukin-6 is a prototypic inflammatory cytokine clearly involved in the development of sporadic CRC and CAC (47). IL-6 is overexpressed in CRC tissues (48, 49) and elevated levels of serum IL-6 correlate with larger tumor size, occurrence of liver metastases, and reduced survival (50). Moreover, increased blood concentration of IL-6 is an independent adverse prognostic marker of survival in CRC patients (51). Patients suffering from inflammatory bowel disease (IBD) – who have an increased risk for developing CAC – show elevated levels of IL-6 in the serum and *lamina propria* (52, 53).

In the AOM/DSS model of CAC, genetic ablation of *Il6* or treatment with anti-IL-6 receptor ameliorated tumor development. This is explained by the fact that IECs critically depend on IL-6 trans-signaling for their survival (54, 55). *Lamina propria* T cells, macrophages, and cancer-associated fibroblasts (CAFs) can all secrete IL-6 in the CRC stroma (53, 55–57). IL-6 was also described to promote angiogenesis (56) and DNA mismatch repair defects (58). In addition, IL-6 directly promotes the accumulation of MDSCs in tumors, thereby facilitating tumor progression (26). Furthermore, MDSC-produced IL-6 limits the development of CD4^+^ T_h_1 cells (59).

Intestinal epithelial cells do not express IL-6R themselves but rely on IL-6 trans-signaling. IL-6 trans-signaling requires soluble IL-6R generated through alternative mRNA splicing of *IL6R* or ADAM17-dependent limited proteolysis of IL-6R on other cells. Soluble IL-6R, then, associates with the ubiquitously expressed glycoprotein 130 (IL-6 ST/GP130) (60). Engagement of IL-6 with its receptor leads to activation of STAT3, which promotes the proliferation of cancer cells and tumor progression (55, 61). Thus, it is not surprising that the level of STAT3 phosphorylation in CRC patients negatively correlates with survival and is therefore indicative of a poor prognosis (62, 63).

## Interleukin-8

In CRC patients, IL-8 expression is upregulated in tumor tissue compared with adjacent healthy colonic tissue (64). Moreover, germline polymorphisms of IL-8 and vascular endothelial growth factor (VEGF), two genes involved in tumor angiogenesis, are associated with increased risk of recurrence in stage III CRC patients treated with adjuvant chemotherapy (65).

Upon AOM/DSS treatment, transgenic mice expressing human *IL8* were found to show increased CRC tumor numbers and load compared with WT counterparts. IL-8 resulted in higher IEC proliferation in these transgenic mice (64).

In humans, the epithelial–mesenchymal transition (EMT) activator protein SNAIL regulates *Il8* expression in CRC stem-like cells (66).

Interleukin-8 mainly acts on myeloid cells to enhance their mobilization (64). These myeloid cells have immune suppressive functions and promote tumor progression (64, 67).

Mechanistically, IL-8 promotes tumor growth, metastasis, chemoresistance, and angiogenesis, as assayed in CRC cell line models (68, 69). IL-8 signals through its receptor CXCR1/2 to activate the Akt and MAPK pathways and promote the expression of genes responsible for cell proliferation, invasion, and angiogenesis (70). Therefore, blockade of IL-8 signaling pathway may represent a promising therapeutic strategy to restrain CRC development.

## Interleukin-9

Interleukin-9 seems to have a dual role for cancer development. Although IL-9 possibly promotes lymphomagenesis (71), it can also inhibit the growth of melanoma either directly or by promoting anti-tumor immunity (72, 73). Compared with healthy individuals, CRC patients showed decreased levels of IL-9 in the serum and intestinal tissues, and IL-9 expression in these samples negatively correlated with tumor progression (74). By contrast, another study found no significant changes in IL-9 expression in serum of CRC patients, with possibly a trend toward higher levels of IL-9 in patients with high-grade tumors (16). Interestingly, in a heterotopic tumor model using the colon carcinoma cell line CT26, *Il9*^−/−^ animals were protected from CRC development and showed better survival compared with challenged WT animals, a phenotype that was T cell-dependent (75).

Various immune cells in the colon have been shown to produce IL-9, such as T cells, dendritic cells, and natural killer (NK) T cells (76–78). Moreover, stromal cells, including CAFs, also produce IL-9 during CRC (79). IL-9 receptor is expressed both on hematopoietic cells and IECs. Therefore, IL-9 may have a direct or indirect mode of action for CRC development (80). Of note, stimulation of human CRC cell lines with IL-9 has also yielded conflicting data. Caco-2 cells showed reduced proliferation and wound closure in the presence of recombinant IL-9 (80), whereas the proliferation of KM12C and KM12SM cell was enhanced upon stimulation with IL-9 (79).

Engagement of the IL-9 receptor leads to STAT5 phosphorylation (80), the expression of which is predictive of poor prognosis and shorter survival in CRC patients (81).

In conclusion, IL-9 may either enhance or inhibit CRC development. Therefore, further studies are required to clearly dissect the role of IL-9 during intestinal tumorigenesis.

## Interleukin-10

In patient samples, IL-10 serum levels increase over time during CRC progression (82, 83), and high preoperative serum levels of IL-10 correlate with poor survival of CRC patients (84). This suggests a tumor-promoting role of IL-10 in CRC patients. In contrast, IL-10 appears to play a protective role in animal models of CRC. IL-10 was required in regulatory T cells (T_reg_) to reduce tumor burden in *Apc*^Min/+^ mice (85). However, during CRC development, T_reg_ cells may switch their cytokine production from IL-10 to IL-17, which promotes tumor development (86). Furthermore, oral administration of IL-10 microparticles decreased polyposis in the *Apc*^Min/+^ model by suppressing the development of IL-17-producing T_reg_ and inducing conventional, IL-17-negative T_reg_ (87). In the *Apc*^Δ468^ model, T cell-restricted ablation of IL-10 increased the number of intestinal polyps by promoting the accumulation of microbes and eosinophils in intestinal tumors (88). In CAC, *Il10*-deficient mice were shown to be more susceptible to spontaneous intestinal tumor development compared with WT animals (89).

In the intestine, a number of cells, including T cells, monocytes, macrophages, and epithelial cells, have been shown to produce IL-10 (90–92). The IL-10 receptor is a heterotetramer complex consisting of two IL-10Rα and two IL-10Rβ molecules. Although IL-10Rα is specific for IL-10, IL-10Rβ is also used for IL-22 and IL-26 signaling. IL-10Rα is constitutively expressed on most hematopoietic cells and colonic IECs (93); yet, it can also be induced on a number of other cells [reviewed in Ref. (94)]. Binding of IL-10 to its receptor activates STAT1, STAT3, and STAT5 (95), and it is not clear which of these pathways is preferentially activated by IL-10 in CRC. This may explain the divergent findings on IL-10 in CRC, and elucidation of the precise role of IL-10 for CRC remains one of the future challenges in the field.

## Interleukin-11

Interleukin-11, a family member of the IL-6 family of cytokines, has recently been implicated in CRC pathogenesis. IL-11 and its receptor have both been shown to be overexpressed in sporadic CRC specimens (96, 97). Moreover, multiple cell types upregulate *IL11* transcript levels during CRC development, including hematopoietic cells, CAFs, and also tumor cells (7, 97). The pro-tumorigenic effect of IL-11 on tumorigenesis was found to be stronger than the one of IL-6, both in models of CAC and sporadic CRC (97). IL-11 has thus emerged as a novel cytokine driving STAT3-dependent intestinal tumorigenesis similarly to IL-6, and further investigation will help to evaluate its role as a diagnostic or therapeutic target for CRC.

## Interleukin-12

Active IL-12 is constituted of two subunits, IL-12p35 and IL-12p40 that are encoded by *IL12A* and *IL12B*, respectively. These subunits may form either an agonistic IL-12p70 heterodimer or an antagonistic IL-12p80 homodimer (98). The IL-12p35 subunit is shared to generate IL-35 (99, 100), whereas IL-12p40 is shared to form IL-23 (101). Therefore, it is difficult to study the effect of IL-12 without interfering at the same time with IL-23 or IL-35 signaling. Nevertheless, in CRC patients high preoperative IL-12p40 serum levels predicted a better survival (102), whereas a low production of IL-12p70 in dendritic cells was associated with a poor prognosis (103).

In mice, the anti-tumor effect of IL-12 has been described in a variety of murine tumor models, including melanoma, sarcoma, renal cell carcinomas, and lymphomas (104–107). Moreover, in a heterotopic tumor model of CRC metastasis, recombinant IL-12 inhibited metastatic events to the lung (105). In the intestine, dendritic cells, macrophages, and B cells have been reported to produce IL-12p35 and IL-12p40. Of note, stimulus by microbial products, such as lipopolysaccharide (LPS) and CpG oligodeoxynucleotide, and also IL-10 are necessary for IL-12 production (108, 109).

Natural killer cells and γδ T cells are the main type of cells expressing IL-12 receptor (110), thus suggesting an indirect action of IL-12 on tumor cells. IL-12 plays a central role both for the induction and the expansion of T_h_1 responses as well as the activation of cytotoxic immune effectors, such as NK and CD8^+^ T cells (111). IL-12 activates and induces IFN-γ production in these cells, which limits tumor growth and metastasis (112–115).

Taken together, these data indicate a protective role of IL-12 in CRC. Although administration of IL-12 as an adjuvant leads to specific immunity against tumor antigens in some patients (116), the net clinical benefit of IL-12 treatment was found to be rather moderate (117, 118). The immunosuppressive effect of the tumor environment and tumor escape mechanisms appear to be the reasons for the failure of the IL-12-based immunotherapy applied so far in human cancers [reviewed in Ref. (119)]. Whether improved administration of IL-12 or combined immunomodulatory approaches that may induce more potent anti-tumor activity remains to be addressed.

## Interleukin-15

Colorectal cancer patients with genomic deletion of *IL15* have a significantly higher risk of tumor recurrence and show reduced survival compared with patients with intact *IL15* (19). Moreover, IL-15 is expressed in human CRC cells *in situ* (120).

In the AOM/DSS model of CAC, *Il15*^−/−^ but not *Il15ra*^−/−^ mice showed higher tumor incidence and tumor size than WT counterparts. Therefore, residual low-affinity IL-15 signaling *via* the shared IL-2Rβ/γc subunits of the IL-15 receptor appears to be able to decrease CAC pathogenesis in *Il15ra*^−/−^ mice. Furthermore, in the same study, loss of IL-15 was found to be associated with an upregulation of inflammatory mediators involved in CRC progression (120). Transgenic mice overexpressing *Il15* better controlled transferred MC-38 colon carcinoma cells and showed no pulmonary metastasis compared with WT mice. Accordingly, in the same model, *Il15*^−/−^ mice showed more rapid tumor growth and died from lung metastases (121). In another study, IL-15 was found to potentiate the effect of immune checkpoint blockade using combined anti-PD-L1 and anti-CTLA-4, thereby leading to prolonged survival in a metastatic murine CT26 CRC model (122). Similarly, IL-15 lead to a synergistic enhancement of agonistic anti-CD40 therapeutic treatment in mouse models of lung metastasis using CT26 or MC38 CRC cells (123). Consequently, IL-15 may have a promising immunotherapeutic potential in the treatment of CRC.

Interleukin-15 is produced by a broad range of cells, including stromal and epithelial cells, and also myeloid cells, such as monocytes, macrophages, and dendritic cells. Dendritic cells, effector CD8^+^ T cells, and NK cells all express the IL-15 receptor that is composed of three distinct receptor chains (124–126). IL-15 can either bind directly to the IL-2Rβ/γc heterodimer with low-affinity or it can be trans-presented to this same heterodimer after binding the high-affinity IL-15Rα subunit (125). Cytotoxic T and NK cells represent the most important immune effectors to integrate the anti-tumorigenic function of IL-15 by activating the APO-1/FAS- or granule-mediated cytotoxic pathway (124, 126). Thus, IL-15 regulates anti-tumor cytotoxicity and modulates the inflammatory tumor microenvironment.

## Interleukin-17

### Interleukin-17A

Of the six members of the IL-17 family identified so far, namely IL-17A, IL-17B, IL-17C, IL-17D, IL-17E, and IL-17F (127), only IL-17A and IL-17F have been studied in greater detail for their contribution to CRC development. Expression of *IL17A* transcripts is enhanced stepwise along the adenoma-to-carcinoma sequence in the stroma and adenomatous/cancerous intestinal epithelium of CRC patients (128). Furthermore, IL-17A serum levels were elevated in CRC patients compared with healthy individuals, positively correlated with tumor size (129) or circulating tumor cells (130), and predicted poor survival (130). Patients with high expression of genes associated with a T_h_17 signature in CRC tissues have a poor prognosis (15, 131).

In mice, *Il17a* deficiency partially protected from CRC in the *Apc*^Min/+^ as well as AOM/DSS models (132, 133). Furthermore, there is probably a link between IL-17A and IL-6 expression, as *Il17a*^−/−^ mice showed markedly reduced IL-6 levels in the AOM/DSS model of CAC (133). Thus, IL-17A may indirectly activate STAT3 through IL-6.

CD4^+^ T_h_17 immune cells are the main source of IL-17A in CRC tumors and adjacent tissues (134, 135); yet, CD8^+^ T cell subsets, γδ T cells, and innate lymphoid cells (ILCs) are also important producers of IL-17A (136–139). IL-17A binds to a heterodimeric receptor comprising an IL-17RA and an IL-17RC chain. The IL-17 receptor is expressed on a variety of cells, such as hematopoietic, fibroblastic, and epithelial cells (140, 141). Although this indicates that engagement of IL-17 receptor signaling may act directly and indirectly on tumor cells, a recent study reports that direct signaling on transformed colonic epithelial cells is sufficient to promote CRC development (140).

### Interleukin-17F

Contrarily to IL-17A, IL-17F, another IL-17 family member, appears to have anti-tumorigenic properties. In a cohort of 102 Tunisian CRC patients, individuals with WT *IL17F* had a longer overall survival compared with patients with polymorphisms in *IL17F* (142). Similarly, the IL17F TT genotype was associated with a lower risk of CRC compared with the TC genotype and C allele in CRC patients from Iran (129). Furthermore, *Il17f*-deficient mice developed more and larger intestinal tumors compared with WT animals in the AOM/DSS model of CAC (143). In a heterotopic tumor model where HCT 116 CRC cells were engineered to overexpress IL-17F, tumor growth was inhibited likely through inhibition of angiogenesis (143). Even though IL-17F is strongly expressed in the colon by activated T cells and colonic epithelial cells (143, 144), IL-17F is downregulated in colorectal tumors (143). As for IL-17A, IL-17F binds to a receptor complex comprising IL-17RA and IL-17RC. Thus, IL-17F may also affect CRC development directly and indirectly.

It is so far still elusive how IL-17A and IL-17F exert such opposing functions through a same receptor. The differential regulation of IL-17A and IL-17F expression during CRC may provide a partial explanation to this paradox. Furthermore, IL-17F has a reduced ability to activate the downstream signaling cascade compared with IL-17A. Finally, IL-17RA and IL-17RC have different binding affinities for IL-17A and IL-17F, which may also account for the different roles of these cytokines [reviewed in Ref. (145)].

Mechanistically, IL-17A activates the NF-κB pathway in CRC cells (146), thereby driving tumor survival and growth [see also tumor necrosis factor (TNF)]. Of note, IL-17F does not lead to the activation of NF-κB in CRC cells, further highlighting the difference between IL-17A and IL-17F signaling (146).

Overall, these studies highlight the differential effect of IL-17A and IL-17F on CRC development. *Il17ra*^−/−^ animals, which are insensitive to IL-17A and IL-17F, show reduced intestinal tumor number, size, and load during sporadic CRC (21). Therefore, the pro-tumorigenic effect of IL-17A is probably dominant over IL-17F in this model. Given different binding affinities of IL-17RA and IL-17RC for IL-17A and IL-17F, it may be relevant to assess CRC development also in *Il17rc*^−/−^animals.

## Interleukin-18

Interleukin-18 is an anti-tumorigenic cytokine. Humans heterozygous for the IL-18 A607C polymorphism exhibit increased risk for CRC development (147). Compared with normal mucosa, IL-18 production is decreased in colon adenocarcinomas. In half of the cases, this reduction in IL-18 expression correlated with lack of *IFNG* and *FASLG* expression in the CRC tissue and the presence of distant metastases (148). Of note, mutations in the IL-18 receptor accessory protein (*IL18RAP*) gene are associated with Crohn’s disease and IBD (149).

Damage of IECs promotes the formation of the NLRP3 inflammasome, which in turn leads to the caspase-1-dependent processing and secretion of active IL-1β and IL-18 (150, 151). Mice deficient in *Il18* or *Il18r1* developed more tumors upon AOM/DSS treatment, compared with WT controls (152). In this model of CAC, IL-18 seems to be particularly important during the early inflammatory phase of the treatment. Indeed, *Il18*^−/−^ mice showed a more severe clinical and histopathological manifestation of colitis than WT animals upon challenge with DSS, a phenotype recapitulated in *Nlrp3*^−/−^ or *Casp1*^−/−^ mice (153).

Murine IECs produce IL-18 under steady state. Secreted IL-18 stimulates IL-18 receptor (IL18-R1) that is expressed by CD4^+^ cells in the *lamina propria*. IL-18 signaling restricts inflammation in the intestine by limiting the differentiation of T_h_17 cells and promoting the expression of effector molecules in T_reg_ (154). In addition, IL-18 is involved in the repair of the intestinal epithelium (155, 156), possibly *via* IL-22 (157), and it downregulates intestinal *Il22bp* expression (157). IL-18 also promotes protective host immunity mediated by cytotoxic cells, including CD8^+^ T lymphocytes, and NK cells (158).

Binding of IL-18 to its receptor IL-18Rα leads to the recruitment of IL-18Rβ to form a high affinity complex that recruits intracellular signaling effectors, including MyD88, IRAK, and TRAF6. As for IL-1 signaling, this cascade eventually results in the activation of NF-κB, JNK, and p38 MAPK (30). Although IL-1 and IL-18 pathways use the same signaling molecules downstream of their respective receptor complexes, they exert opposite function in CRC. This apparent contradiction may be explained by the distinct types of cells each of these cytokines activates within the tumor stroma.

## Interleukin-21

Interleukin-21 appears to have an opposite role for CRC development in humans versus mice. IL-21 is upregulated in patients with ulcerative colitis-associated colon cancer and in the murine model of CAC based on AOM/DSS treatment (159). A systems biology approach aiming at quantifying different immune cell subpopulations *in situ* in human tumor found a positive correlation between IL-21 expression and disease-free survival (131). A preclinical study using IL-21 combined with cetuximab (an anti-EGFR monoclonal antibody) indicated an activation of immune response biomarkers on NK and T cells in stage IV CRC patients, yet it did not evaluate treatment efficacy (160).

Compared with WT controls, reduced tumor size and numbers were found in *Il21*^−/−^ mice treated with AOM/DSS. *Il21*-deficient tumors show higher cell apoptosis and reduced cell proliferation, with high IFNγ and low IL-17 (161). In another study, resistance to CAC in *Il21*^−/−^ mice was associated with reduced CD4^+^ T cell infiltration and decreased production of IL-6 and IL-17A in the intestinal mucosa (159). This suggests a pro-tumorigenic role of IL-21 in the setting of AOM/DSS-induced CAC.

CD4^+^ T helper cells, including T_h_1 and T_h_17 cells, activated NKT cells and T follicular helper cells all can secrete IL-21. IL-21 can act on a broad range of cells, such as B cells, NK cells, activated T cells, dendritic cells, macrophages, fibroblasts, and IECs (159, 162, 163). IL-21 enhances anti-tumor NK and CD8^+^ T cell responses (164, 165). In tumors of AOM/DSS-treated WT mice, *lamina propria* mononuclear cells and tumor-infiltrating immune cells, mostly T and myeloid cells, express the IL-21 receptor (159).

Interleukin-21 signals *via* heterodimers of the IL-21 receptor (IL-21R) and the common cytokine receptor γ-chain (IL-2Rγc). Upon receptor engagement, IL-21 induces the activation of JAK1, JAK3, and mainly STAT3 [reviewed in Ref. (163)].

Taken together, IL-21 has a possible impact on the polarization of the T helper cell response in CRC. Further investigation on the role of IL-21 for CRC may reconcile the current discrepancies from human and mouse studies, e.g., by using animal models of CRC in which inflammation is less central than for the AOM/DSS model.

## Interleukin-22

Interleukin-22 has recently emerged as a novel player in CRC development. In patients, the accumulation of T_h_22 cells is associated with CRC development (166). Furthermore, high levels of IL-22 in the serum or CRC tissue are predictive of a poor survival of patients, and IL-22 promotes resistance to chemotherapy (167). Moreover, the rs1179251 polymorphism in *IL22* gene is associated with an increased risk for CRC (168).

In animal models, IL-22 has been shown to ameliorate experimentally induced colitis and enhance wound healing (169–171). In *Helicobacter hepaticus*/AOM-induced intestinal tumors, IL-22 released by ILCs supported the growth of intestinal tumors in T and B cell-deficient *Rag2*^−/−^ mice (138). *Il22*^−/−^; *Apc*^Min/+^ mice develop smaller tumors than controls. However, deficiency in *Il22* leads to delayed wound healing and increased inflammation and therefore promotes intestinal tumor development in the AOM/DSS model of CAC (157). In addition, animal deficient in *Il22bp*, an antagonist of IL-22 signaling, developed strongly enhanced tumorigenesis. This likely relies on the IL-22BP-dependent regulation of IL-22 activity during intestinal tissue damage and tumorigenesis (157). Subcutaneous injection of primary CRC cells together with anti-IL-22 antibody treatment strongly inhibited tumor development and growth (172).

CD3^+^CD4^+^IL-22^+^ ILCs represent the major source of IL-22 in the intestine (138). IL-22 signaling is transmitted through a receptor complex comprised of the IL-10Rβ and IL-22Rα1 chains. Of note, IL-10Rβ and IL-22Rα1 were recently shown to be upregulated in primary CRC tissue samples (173). IL-22 signaling directly activates STAT3 in epithelial cells and increases stemness and tumorigenic potential in tumor cells through the methyltransferase DOT1L (172). In addition, IL-22 protects CRC cells from chemotherapy *via* STAT3-dependent autocrine secretion of IL-8 (167).

In sum, IL-22 seems to promote CRC development *via* induction of stemness in tumor cells. The mechanisms how IL-22 confers stemness in tumor cells compared with other pro-tumorigenic cytokines also signaling through STAT3, such as IL-6 and IL-11, warrant further investigation. This may be addressed by stratified analysis of the expression of IL-6R or IL-11R compared with IL-22R on intestinal stem cells. Alternatively, the IL-22 signaling is not negatively regulated by suppressor of cytokine signaling 3 (SOCS3), which is the case for IL-6 and IL-11 signaling. Consequently, IL-22 signaling may be more sustained and more pro-tumorigenic compared with other STAT3 activators [reviewed in Ref. (174)].

## Interleukin-23

Bioactive IL-23 is a heterodimeric complex consisting of IL-23p19 (encoded by *IL23A*) and IL-12p40 (encoded by *IL12B*), which are specific for IL-23 or shared with IL-12, respectively. In the serum of CRC patients, IL-23 levels are increased and positively correlate with VEGF (175). In primary CRC tissue, *IL23A* and *IL12B* transcripts are overexpressed, whereas *IL12A* mRNA is not upregulated (176). Moreover, high IL-23 levels together with low SOCS3 expression in primary tumor tissue were predictive of a higher rate of CRC metastasis (177).

*Il23a* deficiency in mice resulted in fewer and smaller tumors compared with WT controls in a model relying on heterozygous loss of *Apc* (21). Furthermore, IL-23 enhanced the metastatic capabilities of SW620 CRC cells after injection into nude mice. Of note, the pro-metastatic effect of IL-23 was dependent on SOCS3, as concomitant overexpression of IL-23 and SOCS3 in SW620 cells reduced the number of metastases (177).

Dendritic cells, macrophages, and neutrophils have been shown to produce IL-23 during intestinal inflammation (178–180). A variety of hematopoietic cells in the intestine can react to IL-23, among them ILCs, T_reg_, and T_h_17 cells. The net biological effect of IL-23 signaling may vary in different cell populations. For instance, IL-23 signaling promotes IL-22 secretion by ILCs and IL-17 production by T_h_17 cells, while it abrogates T_reg_ cell activation (181–183).

Overall, this suggests that IL-23 may indirectly promote tumor cell survival. Indeed, IL-23 has been reported to drive intestinal inflammation by inducing other pro-inflammatory cytokines, such as IL-6, IL-17, and IL-22 (184–186). These cytokines may in turn activate tumor cell proliferation through STAT3 and NF-κB.

## Interleukin-33

Interleukin-33 is a member of the IL-1 family of cytokines, which has recently received greater attention for its contribution to intestinal inflammation and CRC. IL-33 is thought to function as an “alarmin” released upon cellular stress or damage to promote or amplify inflammation (187, 188). Unlike other IL-1 family members, such as IL1-β and IL-18, IL-33 becomes inactivated through caspase-1 cleavage. Cathepsin G and neutrophil elastase have been shown to cleave IL-33 and enhance it bioactivity (189, 190). The role of IL-33 for CRC development and its function in intestinal inflammation are still ambiguous. In IBD patients, expression of IL-33 and its receptor, ST2, positively correlate with the extent of inflammation (191, 192). In animal models of colitis, activation of the IL-33/ST2 pathway either promotes or restrains immunopathology and different phases of intestinal disease (188, 192, 193). Furthermore, IL-33 promotes intestinal T_reg_ function in the inflamed intestine (183). Therefore, the IL-33/ST2 pathway may either promote or inhibit CRC development and was therefore the focus of several recent studies.

In CRC patients, we found overexpression of both IL-33 and ST2 in intestinal adenomas and adenocarcinomas. Although several high-grade adenocarcinomas showed a strong expression of IL-33 and ST2, both proteins were predominately overexpressed in low-grade adenocarcinomas (194). Similar results were reported in a smaller CRC cohort where increased microvessel density of IL-33-positive and ST2-positive tumors was additionally observed (195). Higher amounts of serum IL-33 were measured in CRC patients compared with healthy individuals, thereby further suggesting a tumor-promoting effect of IL-33 (196).

In AOM/DSS-treated mice we could show that intestinal tumor number, size, and grade were markedly reduced in *St2*^−/−^ compared with WT mice (194). Similarly, *Apc*^Min/+^ mice on an *Il33*-deficient background developed fewer and smaller intestinal tumors. In the same study, CRC development was also abrogated by treatment with ST2-blocking antibody (196). This indicates that the nuclear function of IL-33 as a regulator of gene transcription (197) and its role as a soluble cytokine upon secretion (198) may promote CRC pathogenesis in this model. Other studies using heterotopic models suggest that IL-33 signaling may also play a protective role (199) or promote metastasis (200) in CRC.

Epithelial cells and myofibroblasts are the main IL-33-expressing cells in the CRC microenvironment (196, 201, 202). Extracellular IL-33 binds to the IL-33 receptor consisting of ST2 and IL-1 receptor accessory protein (IL-1RAP). ST2, the IL-33-specific subunit of the IL-33 receptor, is expressed on epithelial cells, myofibroblasts, and immune cells (183, 194, 196). Our data suggest that IL-33 does not directly affect the proliferation of tumor cells, but rather decreases the barrier function of the intestine. This in turn allows for increased translocation of bacterial products to normally sterile tissues and induces the production of pro-tumorigenic cytokines, such as IL-6, by immune cells (194). Cytokines, such as IL-6, may then activate STAT3 to promote tumor growth. IL-6 is likely not the only downstream target of IL-33 in CRC. IL-33 stimulation of subepithelial myofibroblasts induced the expression of extracellular matrix components and growth factors associated with intestinal tumor progression (196). Furthermore, IL-33 may foster angiogenesis in CRC (195). As IL-33 and angiogenesis have been linked in previous studies (203, 204), it is conceivable that IL-33 triggers the production of or synergize with pro-angiogenic factors, such as VEGF, which may promote CRC progression and metastasis.

## Granulocyte-Macrophage Colony-Stimulating Factor

Granulocyte-macrophage colony-stimulating factor (GM-CSF) has an ambivalent role for CRC in humans versus mice. GM-CSF expression is elevated in primary colon tumors compared with healthy controls. Moreover, overexpression of GM-CSF and its receptor in intestinal tissue correlates with improved overall survival of CRC patients (205). Interestingly, patient stratification revealed that increased levels of GM-CSF benefit mismatch repair-proficient, yet not repair-deficient patients. This favorable prognostic effect of GM-CSF production by CRC cells was independent from CD16^+^ myeloid and CD8^+^ T cell infiltrations (206).

GM-CSF promotes CAC in AOM/DSS-challenged mice as treatment with a neutralizing anti-GM-CSF antibody decreased tumor development and colitis score in this model (207).

GM-CSF is produced by IECs and even more by neoplastic colonic epithelial cells (206, 207). Commensal microbiota-derived LPS triggers GM-CSF expression in IECs (207). Stromal fibroblasts and lymphocytes adjacent to the CRC tumor have also been found to be positive for GM-CSF (208). More recently, ILC3-derived GM-CSF has been shown to play an important role for intestinal inflammation (209).

Cancerous epithelial cells, monocytes, and antigen-presenting cells all express the GM-CSF receptor in the CRC microenvironment (205–207). GM-CSF induces autocrine or paracrine VEGF release by IECs (207), thereby promoting angiogenesis; yet, it does not have a direct proliferative effect on these cells (208). GM-CSF can act on dendritic cells to promote an anti-tumor response (210) and on monocytes/macrophages to inhibit CRC cell proliferation (206). GM-CSF also recruits and activates eosinophils in the intestine to induce colitis in mice (211), yet high eosinophil counts in human CRC tumor infiltrate are associated with favorable patient outcome (212).

The GM-CSF receptor is a heterodimer consisting of a major binding subunit (GMRα, encoded by *CSF2RA*) and a signaling subunit (βc, encoded by *CSF2RB* and shared with IL-3 and IL-5). GM-CSF binding to its receptor activates the JAK-STAT, the MAPK, and the PI3K pathways, which results in cell survival and proliferation [reviewed in Ref. (213)].

## IFN-γ

IFN-γ expression is reduced in PBMCs of CRC patients (214). There is an association between high serum IFN-γ and absence of nodal metastases in CRC patients (16) and CRC patients with high IFN-γ levels in the supernatant of stimulated PBMC cultures indicate a trend toward better survival in donor CRC patients (215). These data suggest that IFN-γ induces a protective, anti-tumor response in CRC patients. Animal models of CRC support these clinical findings as *Ifng*^−/−^ mice show more and larger intestinal tumors compared with WT controls (35). Moreover, in the sporadic *Apc*^Min/+^ tumor model, heterozygous loss of *Ifng* promoted adenoma progression and induced adenocarcinoma development (216).

Lymphocytes and activated dendritic cells represent the main sources of IFN-γ in the colon (217–219). The IFN-γ receptor (IFNGR) is expressed on almost all nucleated cells, including mature T cells, B cells, macrophages, endothelial, and epithelial tissues (220). IFN-γ signaling is a key determinant for a T_h_1 polarization of immune responses. It enhances MHC class I antigen representation and promotes CD8^+^ T-, NK cell-, and macrophage-mediated cytotoxicity. Hence, IFN-γ robustly stimulates anti-tumor immunity [reviewed in Ref. (221)]. Of note, exogenous IFN-γ inhibited whereas knockdown of *Ifngr1* promoted the growth of HT-29 CRC cells (216).

Mechanistically, IFN-γ acts on CRC cells by inducing STAT1 phosphorylation and inhibiting the EGFR/Erk1/2 and Wnt/β-catenin signaling pathways, thereby restraining cell proliferation (216). Since increased nuclear STAT1 is associated with a better survival in CRC patients (222) and T_h_1 polarization – also induced through IFN-γ – correlates with prolonged survival of CRC patients (15), immunomodulatory approaches selectively inducing IFN-γ production may be considered for CRC therapy. However, IFN-γ can also compromise the barrier function of IEC monolayers *in vitro* (223–225) and possibly alter the colonic epithelial barrier *in vivo* (226). As increased intestinal permeability can drive intestinal inflammation to foster CRC formation (21), approaches promoting IFN-γ production likely represent a balancing act and need to be tightly controlled.

## TGF-β

TGF-β has a dual function during intestinal tumorigenesis. In early tumors, TGF-β is a potent tumor suppressor that induces induced cell cycle arrest (227). The relevance of TGF-β for cancer is corroborated by the frequent occurrence of inactivating mutations found in molecular components of TGF-β signaling (228, 229).

In contrast, high TGF-β levels in the primary tumor or serum correlate with poor survival of CRC patients (7, 230–232). In animal models, a dual function of TGF-β for CRC has also been observed. Loss of TGF-β signaling induced more tumors in AOM-treated mice compared with controls (228). Moreover, heterozygous loss of *Smad4*, a downstream effector molecule of TGF-β receptor, caused larger and more invasive intestinal carcinomas in *Apc*^Δ716/+^ mice (233). This indicates a tumor-suppressive activity of TGF-β in these models. On the other hand, TGF-β was recently shown to promote EMT and metastasis of CRC cell lines *in vivo*, through mechanisms involving SOX4 and miR-1269a (234). Furthermore, TGF-β acting on CAFs promoted the growth of HT-29 cells in a xenograft model of CRC (9). Therefore, TGF-β also has a pro-tumorigenic function. TGF-β and its receptors TGFBR1 and TGFBR2 are commonly expressed on epithelial cells [reviewed in Ref. (235, 236)]. However, other cells in the tumor microenvironment, including CAFs, respond to or secrete TGF-β (237).

Although the exact mechanisms underlying the dual role of TGF-β for CRC have yet to be delineated, a recent study by Calon et al. sheds new light on this paradox. While activation of TGF-β signaling in epithelial cells rapidly induces the expression of cell-cycle checkpoint genes leading to growth arrest [reviewed in Ref. (238)], Calon et al. showed that activation of TGF-β signaling in fibroblasts promotes the metastatic capabilities of intestinal tumor cells (9). Therefore, TGF-β may indirectly exert a pro-tumorigenic effect on CRC cells, *via* the stroma. Indeed, TGF-β may for instance promote IL-11 secretion by CAFs (7), which in turn activates STAT3 and drives the proliferation of tumor cells (97).

## Tumor Necrosis Factor

As indicated by its name, TNF was first identified as an anticancer agent. Yet, it was thereafter recognized as a key cytokine linking inflammation and cancer (239). TNF expression is increased in CRC tissues and TNF serum levels positively correlate with CRC progression and reduced patient survival (240, 241). Accordingly, TNF blockers may possibly reduce the frequency of CAC in treated IBD patients (242–244), yet additional IBD cases are needed to strengthen this initial observation. Another open question remains whether patients with sporadic CRC may also benefit from a therapy based on TNF blockade. In addition to the evaluation of clinical parameters, the efficacy of such a blockade may be also directly assessed *in vivo, via* confocal laser endomicroscopy, as already performed in IBD patients (245).

Hematopoietic cell-produced TNF is critical for intestinal polyp formation in *Apc*^Δ468^ mice, a model of sporadic CRC development (246). Furthermore, TNF blockade strongly diminished tumor development in AOM/DSS-treated mice (247). Similarly, TNF neutralization in obese mice reduced the growth of tumors in a xenograft model using the human CRC line HT-29 (248).

Activated macrophages are the main producers of TNF in CRC (249, 250). TNF signals through TNF receptor 1, which is expressed on most cells, and TNF receptor 2 (TNFR2), which is mainly expressed on hematopoietic cells (251). However, in colitic mice and in IBD patients, TNFR2 expression becomes upregulated on IECs (252). Accordingly, binding of TNF to TNFR2 triggers the proliferation of IECs and CRC cell lines in a STAT3-dependent manner (252, 253) – similar to the IL-6-dependent activation cascade discussed above. In addition, TNF signaling drives the accumulation of MDSCs by promoting their survival (254). Activation of the signaling cascade downstream of the TNF receptors results either in the nuclear translocation of NF-κB and AP-1, which promotes cell survival and proliferation, or induces cell death through caspase activation [reviewed in Ref. (255)].

Most studies in the field of CRC suggest that TNF rather promotes cell survival and thus promotes CRC development. Nevertheless, few reports indicate that TNF may also have an anti-tumor effect in CAC, possibly by providing early antibacterial protection in *Il10*^−/−^ mice (256). In addition, TNF may also serve as a marker of tumor-specific T cells (20). Therefore, the net contribution of TNF to CRC may be determined by the timing of its secretion during tumorigenesis or the type of the immune cells secreting it.

## Vascular Endothelial Growth Factor

Vascular endothelial growth factor is a potent angiogenic factor that is frequently upregulated in cancer where it promotes tumor angiogenesis. In CRC, VEGF expression is elevated in tumor tissue and positively correlates with advanced tumor stage as well as positive lymph node and liver metastasis (257). Furthermore, CRC patients with VEGF-positive tumors show reduced life expectancy (257, 258). VEGF plasma levels are elevated in CRC patients compared with healthy individuals, and high preoperative VEGF plasma levels predict reduced survival (259).

In AOM/DSS-treated mice, both VEGF and its receptor VEGFR2 are strongly upregulated and anti-VEGF treatment reduced tumor growth (260). Antibody-mediated or genetic blockade of VEGF limited tumor growth and increased the survival of *Apc*^Min/+^ mice (261). Inhibition of VEGF receptor signaling similarly reduced intestinal tumor burden in *Apc*^Min/+^ animals (262).

Various cells, such as TAMs, CAFs, tumor cells, platelets, and mast cells, produce VEGF (6). There are several different molecules comprising the VEGF family, namely VEGF-A, VEGF-B, VEGF-C, and VEGF-D, as well as different isoforms of VEGF-A and VEGF-B with potentially different function. Their mode of action has already been reviewed in detail elsewhere (263). Of note, *VEGFA*, *VEGFB*, *VEGFC*, and *VEGFD* expression is modulated during the adenoma–carcinoma sequence in CRC. For instance, *VEGFA* is upregulated in adenomas and carcinomas, whereas *VEGFD* is more abundant in normal tissues (264).

Secreted VEGF binds to either VEGFR1 and VEGFR2 or VGFR3, which are expressed on endothelial cells. In CRC, VEGFR2 is thought to mainly drive angiogenesis to foster tumor development, whereas the function of VEGFR1 is still unclear. However, more recent data reveal that VEGFR1 is expressed on CRC cells and that VEGFR1 signaling actives the β-catenin/Wnt signaling pathway to promote tumor growth (265). In addition, β-catenin/Wnt signaling regulates VEGF expression in CRC (266). Furthermore, engagement of VEGF/VEGFR2 signaling directly in CRC cells leads to STAT3 phosphorylation and promotes tumor development (260).

Taken together, VEGF signaling can support CRC tumor development indirectly, by acting on endothelial cells, to stimulate angiogenesis (267). In addition, VEGF may also directly promote tumor growth through STAT3 and Wnt signaling and represents, therefore, an interesting target for CRC therapy.

## Conclusion – Discussion

Tumors are comprised of tumor cells, immune cells, endothelial cells, and (myo)fibroblasts. These cells form together a microenvironment that determines tumorigenesis and they interact, among others, through the intermediate of cytokines. These cytokines in the tumor stroma critically influence CRC development and progression either by directly stimulating neoplastic epithelial cells or by altering the function or activity of non-tumor cells in the CRC microenvironment. Particular cytokines inhibit, whereas other promote CRC progression (Figure 1). Therefore, it is critical to identify cytokine networks to be either repressed or enhanced using combinatorial therapeutic approaches, as neutralization of individual cytokines may not suffice for therapeutic efficacy (146). Moreover, the net effect of the inflammatory response on the tumor cells may be better predicted by analyzing the molecular signatures downstream of cytokine receptors, rather than solely quantifying individual cytokines. Eventually, further investigations aiming at elucidating the precise reasons for the apparent ambivalent function of certain cytokines are critical, as these studies may also reveal novel mechanisms to be leveraged for therapy.

We discuss below five aspects that we consider to be of particular relevance for the development of future therapeutic strategies for CRC.

### CRC Cancer Types and Cytokine Signatures

Genetic aberrations are necessary for malignancy to be established and to determine the general behavior of the different subtypes of CRC identified so far (268). Of note, differences in the quantity of lesion-infiltrating immune cells are already distinguishable at a very early stage of CRC tumorigenesis, in dependence on subtypes of CRC (269). There is increasing evidence that distinct genetic signatures may be associated with specific cytokine networks. Enterocyte-restricted loss of *Trp53*, which encodes murine p53, is associated with increased intestinal permeability that results in an NF-κB-dependent inflammatory tumor environment (270). Moreover, p53 can negatively regulate IL-6 signaling (271), whereas gain-of-function mutant p53 prolongs TNF-induced NF-κB activation to increase susceptibly to CAC (272). Therefore, inhibition of the IL-6 and TNF pathways may represent an adjuvant therapy specific for CRC with p53 mutations, i.e., CRC subtype with “canonical” molecular signature (CMS2) (268).

As presented earlier, DNA mismatch repair-proficient CRC patients may benefit from a treatment activating the GM-CSF pathway (206), whereas IL-6 blockade may be more suitable in cases of mismatch repair-deficiency (58), i.e., for the subtype of CRC characterized by microsatellite instability (CMS1) (268).

Importantly, several of the studies performed so far using human samples or data from *The Cancer Genome Atlas* may have overlooked the presence of certain cytokines specifically produced by hematopoietic cells, since immune infiltrates are proportionally low in CRC tissues compared to cancerous and non-malignant stromal cells (268). Further research may unveil additional connections between molecular signatures that segregate CRC subtypes (268) and distinct cytokine networks, which may be used for immunomodulatory regimens more specific to tumor subtypes.

### Role of Acute versus Chronic Cytokine-Mediated Inflammation in CRC Tumorigenesis

The pro- versus anti-tumorigenic effect of inflammation is not only determined by the mere presence of define cytokines at a given time in CRC tumors but also by the duration of the induced inflammatory stimulus. Indeed, prolonged or constant exposure to cytokines promotes tumorigenesis, as illustrated by a higher risk for CRC in IBD patients who suffer from chronic intestinal inflammation (273). Chronic inflammation contributes to the generation of MDSCs (274), which are also found in increased frequency in the blood of IBD patients (275). As a matter of fact, CRC tumor-elicited inflammation and MDSC-meditated immunosuppression are closely associated (139). Inflammation-induced accumulation of MDSCs in tumors leads a downregulation of immune surveillance and anti-tumor immunity, thereby facilitating tumor growth (276). Myeloid cells, in particular the so-called TAMs, are mainly responsible for the activation and maintenance of the chronic inflammatory process in tumors. The TAM-induced chronic inflammatory microenvironment may additionally promote genetic instability within the developing tumor epithelial cells [reviewed in Ref. (277–279)].

In contrast to this pro-tumorigenic chronic inflammation, short cytokine exposure or acute inflammatory signals may rather stimulate an anti-tumor response, e.g., by promoting T cell function (280, 281). For instance, treatment with endotoxin was shown to activate lymphocytes and induce an anti-tumor effect in some CRC patients (282). Therefore, therapeutic strategies aiming at changing the pro-tumorigenic chronic inflammation to an acute, antitumor inflammatory state may be beneficial.

### Modulation of Cytokine Networks in the CRC Stroma Using Immune Checkpoint Blockade

Immunomodulation *via* inhibition of CTLA-4 or PD-1/PD-L1 signaling has recently emerged as a potent means for the treatment of malignancies (283). A beneficial effect of CTLA-4/PD-1 blockade has been shown in melanoma, lung, renal, and kidney prostate (283–285). CRC tumors with *RAS* mutation have a low expression of inhibitory molecules and fewer infiltrating immune cells (286). On the other hand, defects in mismatch repair are associated not only with a T_h_1 microenvironment in CRC tumors but also with upregulated expression of multiple immune checkpoints (287). This implies that particular CRC subtypes may show different susceptibility for immune checkpoint therapy. Nevertheless, immune checkpoint blockade for CRC therapy has not yielded promising results so far (288, 289).

### Therapeutic Cytokine Blockade in CRC

Approaches targeting selected cytokine pathways or networks may directly restrain CRC tumorigenesis or improve the response rate of CRC tumors to chemotherapies or checkpoint inhibitors. As a matter of fact, clinical trials are completed or ongoing to evaluate the effect of blocking cytokines, such as IL-1α, IL-1β, IL-6, IL-10, IL-21, TNF, or VEGF. In spite of the above-presented multiple function of VEGF signaling in CRC, therapeutic antibody-mediated blockade of VEGF-A (using bevacizumab) did not prolong the survival of stages II and III CRC patients when given in combination with chemotherapy (290). Nevertheless, treatment with the same VEGF-A blocking antibody (bevacizumab) in combination with a different chemotherapy improved the outcome in patients with metastatic CRC (291). Furthermore, a phase I/II trial and a phase III trial have been completed in patients with solid malignancies to evaluate a blockade of IL-6 or TNF, respectively. Unfortunately, both trials showed little to no effect in disease control (292, 293). Indeed, inhibition of single cytokines will probably not yield strong results as cytokine signals are often overlapping. For instance, IL-6 exerts its pro-tumorigenic function through STAT3, a signaling pathway also switched on by multiple other cytokines, such as IL-11, IL-21, or IL-22. Therefore, combinatorial approaches targeting multiple cytokines or their downstream signaling molecules, also conjointly with chemotherapy or immune checkpoint blockade, may prove to be more effective. Such approaches may possibly also permit a lower treatment dose of checkpoint inhibitors, thereby reducing the risk for immune-related adverse events (294, 295). Such type of therapeutic strategy has actually already shown increased anti-tumor activity in a murine model of metastatic CRC (122).

### Manipulation of the Microbiota

Manipulation of the microbiota may represent an additional future therapeutic strategy for the treatment of CRC. Overall changes in microbial communities have been found in the adenoma-to-carcinoma sequence of CRC, compared with healthy controls (296). Different bacterial communities are either under- or overrepresented in cancerous versus adjacent non-cancerous intestinal tissues (297). Importantly, certain types of commensal bacteria can influence the host immune system by promoting the accumulation of T_h_17 or T_reg_ in the intestinal mucosa (298, 299). In addition, commensal and pathogenic bacteria species induce different cytokine responses in IECs (300, 301). Alterations in the microbiota have been shown to drive IL-17C production from IECs to promote tumorigenesis (302). Intestinal tumors are frequently covered by microbial biofilms, which correlates with enhanced epithelial cell IL-6 and STAT3 activation (303). Moreover, bacteria-derived butyrate induces IL-18 in the colonic epithelium (304). Finally, the intestinal microbiota can influence the outcome of tumor immunotherapy, possibly by augmenting dendritic cell function and subsequent priming of anti-tumor T cells (305, 306). Therefore, it is conceivable that modulation of the microbiome may permit to influence the composition of the immune effectors and the cytokine networks within the CRC stroma, which may be used as adjuvant for therapy. A future objective in the field will, thus, be to advance our understanding on how microbes cross talk with the host to either promote or inhibit CRC formation. Improved knowledge of such communication pathways will likely ameliorate the treatment of CRC.

## Author Contributions

PK: conception and writing of the manuscript; LM: writing of the manuscript; MHW: writing of the manuscript; and TR: critical review of the manuscript.

## Conflict of Interest Statement

The authors declare that the research was conducted in the absence of any commercial or financial relationships that could be construed as a potential conflict of interest.

## Abbreviations

AOM, azoxymethane; CAC, colitis-associated (colorectal) cancer; CAF, cancer-associated fibroblasts; CRC, colorectal cancer; DSS, dextran sulfate sodium; IBD, inflammatory bowel disease; IEC, intestinal epithelial cell; IL, interleukin; ILC(s), innate lymphoid cell(s); LPS, lipopolysaccharide; MDSCs, myeloid-derived suppressor cells; T_h_, CD4^+^ T helper cell; TNF, tumor necrosis factor; T_reg_, regulatory T cell(s); WT, wild-type.
